# Effect of Crystal Structure Anisotropy on the Corrosion Characteristics of Metals in Liquid Lead: A Molecular Dynamics Simulation Study

**DOI:** 10.3390/ma18235396

**Published:** 2025-11-30

**Authors:** Na Liang, Bin Long, Zhangshun Ruan, Xiaogang Fu, Xusheng Zhang, Yajie He, Shenghui Lu, Lingzhi Chen

**Affiliations:** Reactor Engineering Technology Research Institute, China Institute of Atomic Energy, Beijing 102413, China

**Keywords:** crystal structure, corrosion, lead, anisotropy, molecular dynamics simulation

## Abstract

This study investigated the compatibility of lead with distinct crystal planes of Fe with a body-centered cubic (bcc) crystal structure and Ni with a face-centered cubic (fcc) crystal structure using molecular dynamics (MD) simulation. It was found that corrosion anisotropy depends mainly on the role of different crystal planes in regulating the spatial distribution of liquid lead. The essence of this regulation can be attributed to the interaction between the crystal plane and the liquid lead atoms. In consequence of the periodic arrangement of the crystal planes, the close-packed plane exhibits the highest atomic density and the widest interplanar distance. This configuration minimizes the interaction of the liquid lead atoms with the other crystal planes, thereby maximizing the regulatory effect on the distribution of the liquid lead atoms. The regulatory effect results in the formation of a regular layer-like distribution of the lead atoms, with a spacing between layers that is analogous to the crystal planes. This distribution mechanism effectively prevents the dissolution of atoms on the crystal surface into the liquid lead side by separating the atoms of the solid–liquid system from each other. Accordingly, for pure metals with a bcc crystal structure, corrosion resistance anisotropy indicates that the (111) plane is the most susceptible to corrosion, followed by the (001) plane, and the close-packed plane of (110) exhibits the most corrosion-resistant properties. As for fcc crystals, the corrosion resistance of the distinct planes is ordered as follows: (111) > (001) > (110).

## 1. Introduction

Lead (Pb) and lead–bismuth eutectic (LBE: 44.5 wt.% Pb + 55.5 wt.% Bi) are considered, at present, potential candidates for the coolant of new-generation fast-spectrum nuclear reactors and accelerated-driven systems (ADSs) and for liquid spallation neutron sources. The lead-cooled fast reactor (LFR) operates on a fast-neutron spectrum, where the fission heat is efficiently removed by a liquid lead–bismuth coolant, and is often coupled with a closed fuel cycle to achieve efficient fuel utilization and breeding. However, the main problem of these heavy Liquid metals (HLMs) is a rather high corrosion rate of steels at high temperatures (>773 K~500 °C) [1,2,3,4,5,6]. The high dissolution of alloying elements in liquid lead causes the destruction of materials. Therefore, severe corrosion problems of structural materials have become a bottleneck limiting the development of lead-cooled fast reactors (LFRs) [7,8,9,10].

The corrosion mechanism of nuclear reactor structural materials in liquid lead/lead–bismuth involves a variety of physicochemical processes [11]. The corrosion effect of liquid metals on materials is dependent on several factors, including the temperature of the corrosive medium, the concentration of dissolved oxygen, the flow rate, and the degree of wetting of the materials. Therefore, the main corrosion mechanisms involved are oxidizing corrosion, dissolving corrosion, and erosion corrosion [2,12,13,14,15,16]. Among them, the high dissolution of alloying elements in liquid lead leads to severe mass migration of the materials and thus reduces the mechanical loading performance of the components, severely limiting the lifetime of reactor operation [17].

Much research has been reported on the study of dissolution corrosion, particularly by using molecular dynamics simulations. Alan Maulana et al. investigated the corrosion phenomena of steels in liquid LBE by molecular dynamics methods and calculated the diffusion depth of Pb atoms in Fe-10%Ni-16%Cr, Fe-10%Ni, and Fe-16%Cr [18]. Artoto Arkundato et al. investigated the effect of oxygen injection into lead coolant to reduce the corrosion rate of iron by evaluating the mean square displacement curves, radial distribution function curves, and concluded that the injection of 1% oxygen atoms into lead can reduce the corrosion rate to the level of 2.09% [19]. In addition, Yun Gao et al. further investigated the diffusion properties of metallic impurity in the LBE. They found that the experimental and simulation results can be reconciled well by assuming the metallic impurities diffuse mainly as nanoscopic clusters below 1300 K, and diffuse in single-atom form at higher temperatures [20]. Furthermore, other liquid–solid and solid–solid interdiffusion related to the evolution of interfacial properties have been extensively studied [21,22,23,24,25,26]. These studies have attempted to characterize and model the relevant diffusion problems from a microscopic point of view by using molecular dynamics simulations and have provided an atomic-scale understanding of the diffusion phenomena. The key crystallographic parameters of the two crystal structures are summarized in Table 1.

Despite the many great advances in the field of interfacial diffusion, including numerous studies on the corrosion characterization of crystalline anisotropy in high-temperature liquids [27,28,29,30,31], few comprehensive studies have been carried out to focus on the factors responsible for the corrosion anisotropy on distinct crystal planes of different crystal structures and to summarize a general rule. In this work, we have calculated the corrosion properties of different crystal planes of bcc and fcc crystals in a high-temperature liquid corrosion environment. By analyzing the corrosion properties of different planes, a general law that generates corrosion anisotropy is derived. In addition, the mechanism of corrosion anisotropy at the atomic scale is also analyzed. The findings of this study can be employed for microstructure design and selection of new corrosion-resistant materials.

## 2. Models and Methods

All simulations were performed using the Large-scale Atomic/Molecular Massively Parallel Simulator (LAMMPS) [32], distributed by Sandia Labs (Albuquerque, NM, USA). The timestep of the MD simulation was Δt = 1 fs. Periodic conditions were applied for all models. The Nose-Hoover thermostat was used to control the temperature and pressure [33]. The embedded atom method (EAM) potential was used to describe Fe-Fe [34], Ni-Ni [35], and Pb-Pb [36], while the Lennard-Jones potential was used for Fe-Pb [37] and Ni-Pb [37], respectively.

The solid–liquid models of Fe-Pb were constructed by the (001), (110), and (111) planes of Fe single crystals and liquid Pb samples. The relevant parameters of the models are listed in Table 2. The solid–liquid interface was conducted as follows. Initially, Fe single crystals with distinct crystallographic orientations (001), (110), and (111) were equilibrated for 50 ps using the NPT ensemble to prepare the cross-sectional areas of the interfaces at 673 K. Then, the solid Pb model was equilibrated for 50 ps at 601 K (the melting point of Pb) using the NP_Y_AT ensemble, in which the model of liquid Pb is only allowed to fluctuate along the direction of the *y*-axis, and fixed in the cross-sectional area (xz). The equilibrated Fe single crystal model and the Pb model were assembled with a gap of 3 Å between them. The simulations of the corrosion process were conducted by subjecting the assembled solid–liquid interface model to a longer equilibrium relaxation period at elevated temperatures. In this study, the corrosion process was simulated by equilibrating for 2 ns under the NP_Y_AT ensemble and then relaxing for 50 ps under the NVT ensemble to calculate the fine-scale density profiles. The analogous procedures were also performed for the Ni-Pb solid–liquid interfaces model.

## 3. Results

### 3.1. Diffusion Characteristics of the Fe-Pb Solid–Liquid Interface

**Figure 1:**
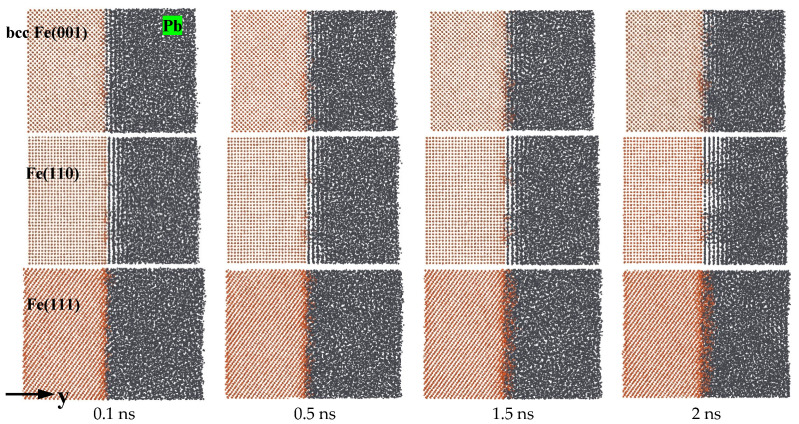
Atomic diffusion processes of Fe(001)-Pb, Fe(110)-Pb and Fe(111)-Pb at 673 K.

Figure 1 depicts the atomic diffusion behaviors of the equilibrated Fe(001)-Pb, Fe(110)-Pb, and Fe(111)-Pb solid–liquid interfaces at a temperature of T = 673 K. The snapshots were captured at 0.1 ns, 0.5 ns, 1.5 ns, and 2 ns, respectively. It can be observed that the phenomenon of atomic diffusion is accelerated with the extension of corrosion time on different crystal planes. However, the interfacial diffusion properties differ, indicating the presence of corrosion anisotropy. In the case of Fe(110), the pronounced ordering of liquid Pb is visible throughout the simulation time (2 ns). The ordering distribution of liquid Pb in contact with Fe(001) was relatively low, while the Fe(111) was free of ordering throughout the corrosion process. The diffusion of Fe atoms into liquid Pb occurred on all three crystal planes, indicating that dissolution corrosion occurred on all planes. Notably, the dissolution corrosion on the Fe(111) plane was significantly more pronounced than that observed on the Fe(110) and Fe(001) planes, and a uniform dissolution layer of Fe atoms appeared at the solid–liquid interface at 0.1 ns. Furthermore, the dissolution and diffusion of Fe atoms into the liquid Pb also occurred at the (110) and (001) planes, yet to a significantly lesser extent. In comparison, the (110) planes demonstrate the most robust resistance to dissolution corrosion, largely due to the pronounced interfacial delamination phenomenon, which facilitates a more pronounced degree of solid–liquid separation. The results of the calculations thus indicate that for Fe of bcc, dissolution corrosion was most susceptible to occur in the (111) plane, followed by the (001) plane, and that the (110) plane exhibited the most resistance to corrosion by liquid Pb.

**Figure 2:**
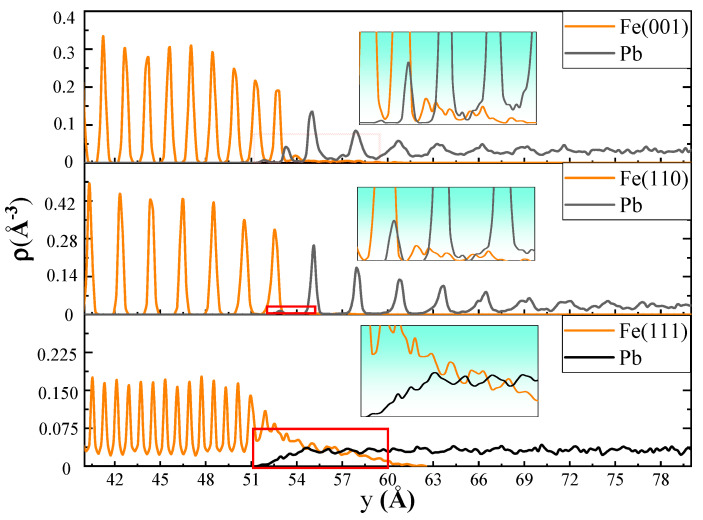
The fine-scale density profiles of the Fe(001)-Pb, Fe(110)-Pb, and Fe(111)-Pb at the end of the diffusion at 673 K.

The snapshots at each moment permitted visualization of the high-temperature diffusion process at the solid–liquid interfaces. To further refine the analysis of the diffusion behavior of the atoms at the solid–liquid interface, fine-scale density profiles were calculated for the three crystal models. To obtain the fine-scale density profiles, the assembled models were sliced into a number of thin bins along the *y*-axis; the bin size was chosen to be 1/40th of the lattice constant. Figure 2 shows fine-scale density profiles of the Fe-Pb models with three crystal planes at 673 K. It can be observed that the densities of atoms at different crystal planes of the Fe bulk are arranged in a periodic pattern. The (110) crystal plane exhibits the widest interplanar distance and the loosest arrangement of density peaks. Followed by (001), and the density peaks are most tightly arranged in the (111) plane. It is notable that the density distribution of liquid Pb atoms at approximately 18 Å near the (110) and (001) planes also exhibits a distinct pattern of periodic peak distribution. As the distance from the crystal plane increases, the amplitude of the peaks gradually decreases until their disappearance, and the density distribution of Pb reverts to the typical liquid disorder. In addition, the density peak width, peak spacing, and oscillation period of the liquid Pb in contact with the (110) plane are nearly identical to those of the (110) plane itself. Furthermore, the crystal distribution characteristics are more pronounced in this area relative to those observed in the liquid Pb in contact with the (001) plane. Meanwhile, the density distribution of liquid Pb in contact with the (111) plane does not exhibit a periodic peak pattern. Instead, it clearly shows the mutual diffusion of Fe and Pb atoms at the solid–liquid interface. This suggests the presence of interfacial alloying, which is characterized by severe dissolution corrosion at the interface. Overlapping of the density peaks of Fe and Pb atoms also occurs at the (110) and (001) planes. However, the (110) plane demonstrates superior structural integrity, with less Fe diffusing into the liquid Pb and a shorter diffusion distance. The alloying layer is primarily the result of the dissolution of Fe atoms into liquid Pb. Due to the larger atomic radius of Pb (0.175 nm, 0.126 for Fe, and 0.124 for Ni), it is challenging for Pb atoms to penetrate the Fe crystal lattice without disrupting its structure. Therefore, the diffusion of Pb atoms into the Fe crystal occurs only when a sufficient quantity of Fe atoms is dissolved and the crystal surface contains a high density of defects.

**Figure 3:**
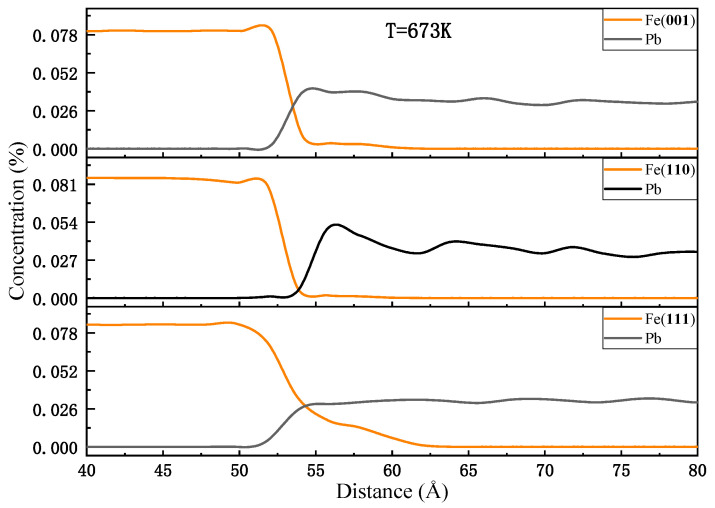
Concentration profiles at the end of the diffusion at 673 K.

Figure 3 illustrates the variation in the number of Fe and Pb atoms as a function of position along the diffusion direction. The three models exhibit distinct diffusion characteristics. For the (001) and (110) planes, the depth of the Fe diffusion layer into liquid Pb is comparable, approximately 5 Å. However, the distance between the Fe bulk of the (110) plane and liquid Pb is greater, and the Pb concentration near the interface is slightly higher than that away from the interface. In contrast, the distance between the Fe of the (001) plane and the liquid Pb is closer, which can be supported by Figure 1 and Figure 2. It indicates that the number of Pb atoms diffusing into the diffusion layer of Fe atoms in the (110) plane is low, while that number is high in the (001) plane. The Fe(111)-Pb interface exhibits a severe dissolution of Fe atoms, with the depth of the diffusion layer of Fe atoms into liquid Pb estimated to be approximately 15 Å. Additionally, the amount of liquid Pb appears to be more uniform throughout the diffusion direction. The red rectangle marks the region magnified below to show atomic details at the interface.

### 3.2. Diffusion Characteristics of the Ni-Pb Solid–Liquid Interface

**Figure 4:**
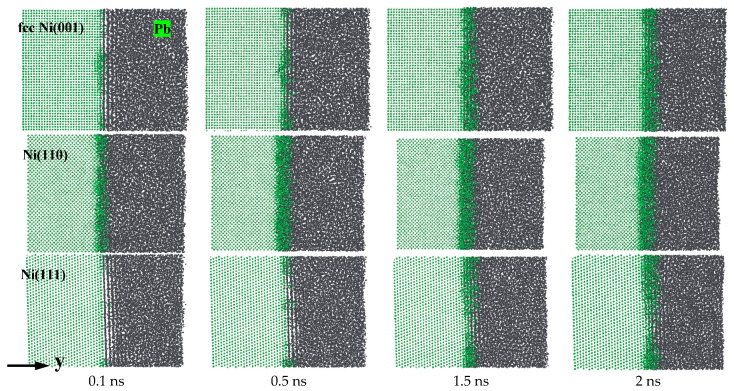
Atomic diffusion processes of Ni(001)-Pb, Ni(110)-Pb, and Ni(111)-Pb at 673 K.

Figure 4 shows the snapshots of representative diffusion processes for Ni (001), (110), and (111)-Pb interfaces at T = 673 K. They were captured at 0.1 ns, 0.5 ns, 1.5 ns, and 2 ns, respectively. It can be observed intuitively that the diffusion of Ni atoms into liquid Pb is intensified with the increase in simulation time, especially for the (001) and (110) planes of Ni. The thickness of the diffusion layer varies significantly, which suggests that Ni with fcc crystal structure also exhibits anisotropy of dissolution corrosion in liquid Pb. The Ni(111)-Pb interface exhibited the least amount of diffusion of Ni atoms into liquid Pb, followed by the Ni(001)-Pb interface. In contrast, at the Ni(110)-Pb interface, severe diffusion of Ni atoms occurred, yet the growth of the thickness of the diffusion layer slowed down with the extension of the simulation time. By examining the trajectory files of the diffusion process, it can be observed that when the diffusion layer reaches a certain thickness, the number of Ni atoms dissolved on the side of the diffusion layer closest to the solid Ni is significantly higher than the number of Pb atoms. The large number of Ni atoms wrapped around the near surface of the solid Ni prevents the Pb atoms from interacting frequently with the surface of Ni. Consequently, the number of Pb atoms directly in contact with the solid Ni decreases compared to the initial moments. This results in a reduction in the observed dissolution rate of the solid Ni and a deceleration of the growth of the diffusion layer. Notably, the liquid Pb distribution at the Ni(001)-Pb and Ni(111)-Pb interfaces appears to be regularly stratified when viewed from the direction of the vertical *x*-axis, allowing the liquid Pb and solid Ni to separate from each other. As the solid Ni bulks are gradually corroded by liquid Pb, the layered structure of the liquid Pb is gradually covered by the filling of the diffusion layer, while no liquid Pb was observed to lie at the Ni(110)-Pb interface during the simulation time. The snapshots of the diffusion process demonstrate that Ni with fcc crystal structure exhibits the lowest dissolution rate at the (111) plane, followed by the (001) plane. In contrast, the (110) plane is the most vulnerable to corrosion by liquid Pb.

**Figure 5:**
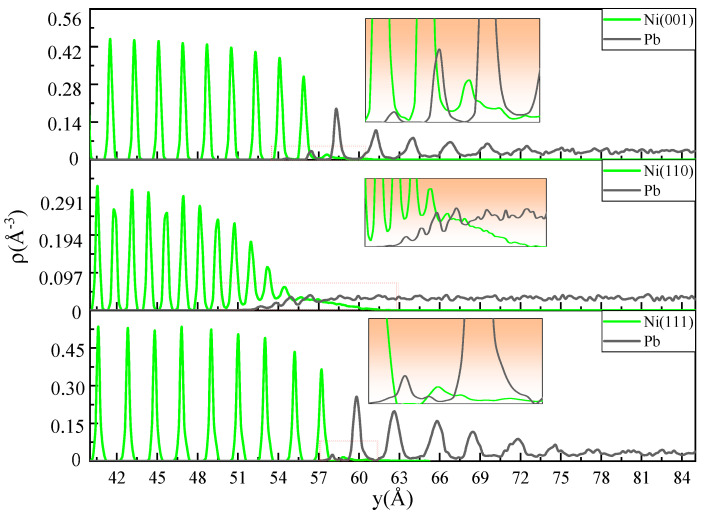
The fine-scale density profiles of the Ni(001)-Pb, Ni(110)-Pb, and Ni(111)-Pb at 0.1 ns at 673 K.

Figure 5 depicts the fine-scale density profiles of the Ni-Pb models with three crystal planes at 673 K for a simulation time of 0.1 ns. It can be observed that the atomic density distributions of Ni single crystals with different crystal planes all exhibit typical periodic peak arrangements, except that the height and peak distance of the peaks of different crystal planes are different (the heights of the peaks as follow: (110) plane: peak height = 1, d-spacing = 4, (001) plane: peak height = 2, d-spacing = 5, (111) plane: peak height = 3, d-spacing = 6). For the (111) plane, the peak distance is the widest, and the peaks have the highest amplitude of the three. The (001) plane has the next highest density peak amplitude, while the (110) plane has the lowest density peak amplitude, and the peaks are the most closely aligned. Therefore, the fine-scale density profiles characterize the arrangement of the various crystal planes of the crystal structure. Notably, the liquid Pb adjacent to the three crystal planes exhibits distinct structural characteristics. In the Ni(111)-Pb model, the liquid Pb exhibits an evident periodic density peak arrangement in the first six layers. As the distance from the solid–liquid interface increases, the amplitude of the peaks gradually diminishes until they disappear, transforming into the typical disorder-like distribution of liquid atoms. The distance of the density peaks in the first and second layers is comparable to that observed on the (111) plane. It can be inferred from the figure that Pb atoms have diffused into the (111) plane of Ni bulk. This is due to the detachment of Ni atoms from the original crystal surface, which results in the formation of vacancy defects within the (111) plane. This subsequently allows Pb atoms to diffuse along the defects into the interior of the Ni single crystal. However, the number of Ni and Pb atoms involved in interdiffusion is relatively limited, as indicated by the density map. The (001) plane is analogous to the (111) plane, and the liquid Pb adjacent to it also exhibits a periodic arrangement of density peaks. The amplitude of these peaks diminishes with increasing distance from the interface until they disappear. The amplitude of the peaks is relatively low, and the number of Pb atoms diffusing into the (001) plane is higher than that observed for the (111) plane. This indicates that the number of Ni atoms dissolved from the (001) plane to the liquid Pb side is relatively high. For the Ni(110)-Pb model, the most serious dissolution of Ni atoms to the liquid Pb side occurs at the interface. This process results in the destruction of the structural integrity of the (110) plane, thereby facilitating the diffusion of a considerable number of Pb atoms into the Ni lattice. The interdiffusion layer can attain a thickness of 11.8 Å, which suggests that dissolution corrosion occurring at the (110) plane is the most significant. Furthermore, the atomic density distribution of liquid Pb in close proximity to this plane is most consistent with the typical liquid distribution pattern, namely the disorder-like distribution.

## 4. Discussion

### 4.1. Comparative Analysis of Corrosion Anisotropy

**Figure 6:**
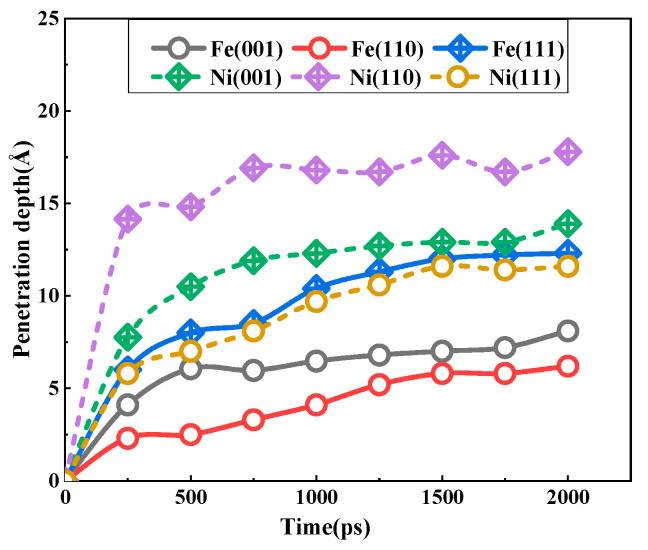
The time-dependent penetration depths of Fe and Ni atoms for the three interfaces at 673 K.

Figure 6 depicts the diffusion depth of bcc iron and fcc nickel atoms on the low-index crystal plane over a 2 ns simulation time. It can be observed that the diffusion depth is obviously deepened with the increase in time. Furthermore, the growth rate of each curve exhibits a consistent trend. The fastest increase is observed during the initial 250 ps, followed by a gradual deceleration in the subsequent 250–500 ps interval. The slowest growth rate is evident during the 500–2000 ps period. As previously discussed, the increase in corrosion time resulted in the aggregation of Fe, Ni, and Pb atoms at the solid–liquid interface, forming a diffusion layer that grew in thickness over time. This diffusion layer, situated in proximity to the solid side, exhibited two distinct characteristics: firstly, it continued to undergo diffusion, and secondly, it served as a barrier, preventing a large influx of Pb atoms from surging to the solid single crystal. Consequently, the dissolution rate is at its maximum at the initial stage of solid–liquid interfacial contact. As the diffusion layer increases, the dissolution rate declines, and the growth rate of the diffusion depth slows down. As illustrated in Figure 6, for bcc Fe, the Fe atomic penetration depth of the (111) plane is the greatest among the three planes, with a value of approximately 12.3 Å. The depth values for the (001) and (110) planes are 8.1 Å and 6.2 Å, respectively. With regard to the fcc Ni, the most significant dissolution corrosion phenomenon is observed at the (110) plane, with a depth of approximately 17.8 Å. This is followed by the (001) plane, which exhibits a depth of 13.9 Å, and the (111) plane, which displays a depth of 11.6 Å.

From the above part of the data discussion on the interfacial diffusion properties of each crystal plane of Fe and Ni in liquid Pb, it can be concluded that for Fe with a bcc crystal structure, the corrosion resistance of the three crystal planes is ranked as (110) > (001) > (111), while for Ni with fcc crystal structure, it is ranked as (111) > (001) > (110). In addition, the other literature that has focused on solid–liquid interfacial diffusion properties [28,29,30,31] also showed that materials with bcc and fcc crystal structures have the same corrosion-resistant anisotropy characteristics as the above calculations. It appears that the corrosion resistance anisotropy is somehow related to the densely arranged crystal planes. In other words, the denser the crystal planes, the greater the corrosion resistance. And a more comprehensive examination of the distribution characteristics of the liquid Pb reveals that the Pb atoms adjacent to the densest planes (110) and (111) of the bcc and fcc crystal structures have exhibited pronounced ordering. In a periodically arranged crystal structure, the interplanar distance between the densest planes is the largest. As the interplanar distance increases, the degree of delamination of the liquid Pb adjacent to it also increases. As illustrated in Figure 2 and Figure 5, the distances of the periodic density peaks presented by several layers of lead atoms near the interfaces of the Fe(110)-Pb and Ni(111)-Pb models are very similar to the density peak distances of the neighboring crystal planes, which to some extent reflects the tuning of the spatial distribution of the liquid lead atoms by the interplanar distance of the solid crystal.

### 4.2. Corrosion Anisotropy Mechanism Analysis

**Figure 7:**
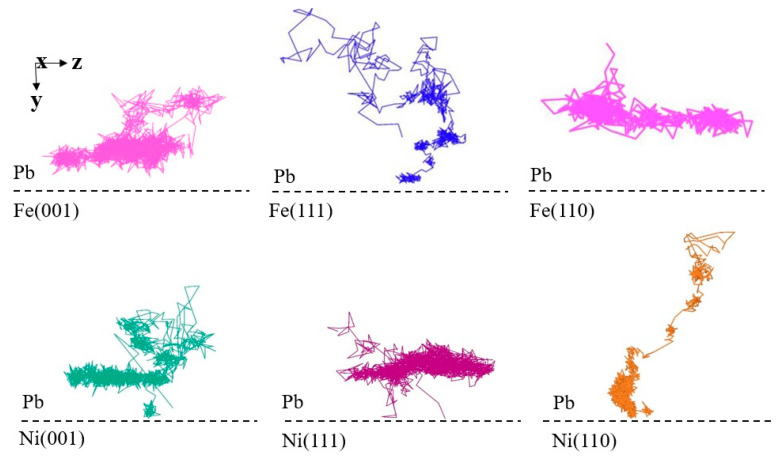
Typical diffusion trajectories of Pb atoms at the solid–liquid interfaces.

Table 3 shows the values of the interplanar distance for bcc and fcc crystal structures. It can be observed that value and corrosion resistance exhibit a positive correlation, with the larger the interplanar distance, the stronger the corrosion resistance of the plane. The mechanism by which the interplanar distance affects the corrosion resistance of the material can be observed by examining the trajectories of the atoms during the diffusion process (Figure 7). The typical trajectories of liquid Pb atoms near the planes with larger interplanar distance are random motions in the direction parallel to the solid–liquid interface. In contrast, the typical trajectories of liquid Pb atoms in contact with the planes with a smaller interplanar distance tend to be random motions in the direction perpendicular to the solid–liquid interface. This diffusion process elucidates the underlying mechanism responsible for the observed differences in corrosion resistance of different crystal surfaces at the atomic scale.

The different trajectories of the liquid Pb can be attributed to the tuning effect of the spatial distribution of the Pb atoms by the different crystal structures, which is manifested in the layering of the liquid lead at the close-packed planes. The obvious layering phenomenon of Pb leads to the separation of liquid Pb and solid crystals from each other, which effectively prevents the dissolution of Fe and Ni atoms into the liquid Pb side. For the (111) and (110) crystal planes, which are the most loosely arranged atoms in the crystal structures of bcc and fcc, respectively, there is no delamination of the adjacent liquid Pb at the interface. The influence of planes with the lowest surface density in regulating the ordering of the spatial distribution of liquid Pb is comparatively limited, primarily due to the lower atomic density of these crystalline planes. This results in Pb atoms interacting with the other two crystal planes, leading to multi-directional interactions that partially offset the single-directional interactions. Consequently, the liquid Pb atoms surrounding the plane with low surface density exhibit a disordered spatial distribution, which is characteristic of the liquid state. This results in a closer proximity of dissimilar atoms at the solid–liquid interface and stronger interactions, which in turn leads to more severe dissolution corrosion occurring at the interface in a shorter time. It can be concluded that the tuning effect of crystal structure anisotropy on the distribution of liquid Pb atoms at the interface plays an important role in the corrosion resistance of the crystal itself.

**Figure 8:**
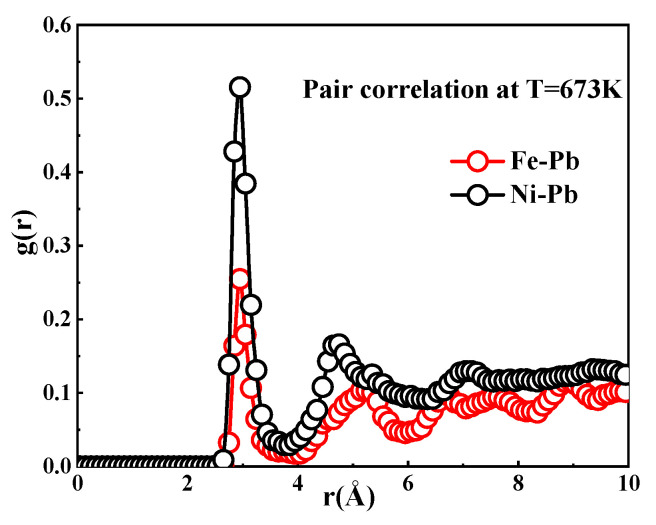
Pair distribution function involving Fe and Ni atoms within Pb at 673 K.

In addition, the corrosion resistance of a material is closely related to the type of element, in addition to the crystal structure. Figure 8 shows the pair distribution functions [22] of Fe and Ni atoms in liquid Pb at 673 K, respectively. The curves describe the structuring of the liquid Pb around these atoms. The coordination numbers of Ni are higher than those of Fe, as demonstrated by the integration of the first peaks in the curves. Therefore, it can be inferred that the element Ni is more susceptible to dissolution in Pb than the element Fe. These data demonstrate that the corrosion of materials is the result of a combination of the intrinsic structure of the material and the specific chemical composition of the elements present.

## 5. Conclusions

To investigate the effect of crystal structure anisotropy on the corrosion resistance to liquid Pb, the compatibility of Pb with different crystal planes of Fe with bcc crystal structure and Ni with fcc crystal structure was investigated. The calculated results indicate that for pure metals with a bcc crystal structure, corrosion is most vulnerable to occur at the (111) plane, followed by the (001) plane. And the (110) plane was found to exhibit the greatest resistance to corrosion, while for pure metals with fcc crystal structure, corrosion most easily occurs at the (110) plane, followed by the (001) plane. The (111) plane is the most corrosion resistant. The commonality between the corrosion-resistant anisotropies of the two crystal structures lies in the ordered hierarchical distribution of the liquid lead atoms at the interface of the surface with higher density. This distribution feature separates the atoms of the solid–liquid system from each other, reduces the interaction of the solid–liquid atoms, and thus enhances the corrosion resistance of the crystalline materials. The role of the plane with lower density in regulating the spatial distribution of liquid Pb is weak, primarily because when the atomic density of the crystal plane is low, the Pb atoms are subjected to interactions with other planes simultaneously. This results in a multidirectional cancelation of effects, which ultimately leads to an irregular spatial distribution of liquid Pb at the interface. Concurrently, the random motion of Pb atoms results in frequent impacts on the crystal plane, making it easier for atoms within the crystal plane to diffuse to the liquid Pb side.

Consequently, the densest and most widely spaced crystal planes can play a greater role in tuning the liquid Pb atoms in the direction perpendicular to the crystal planes, allowing them to maintain the crystal’s own lattice properties at a short distance. This results in the most effective spatial regulation of the liquid Pb, which in turn exhibits the best corrosion resistance. This conclusion will provide new solutions for the selection of corrosion-resistant materials and the design of new corrosion-resistant materials.

## Figures and Tables

**Table 1:** Key Crystallographic Parameters of the BCC and FCC Metals Investigated in this Study.

Parameter	BCC	FCC
Space Group	Im-3m (No. 229)	Fm-3m (No. 225)
Typical Elements	Fe (α-Fe)	Ni
Atoms per Unit cell	2	4
Atomic Coordinates	(0, 0, 0), (½, ½, ½)	(0, 0, 0), (½, ½, 0), (½, 0, ½), (0, ½, ½)
Atomic Packing Factor	0.68	0.74
Closed-Packed Plane	{110}	{111}
Typical Lattice Parameter	α-Fe: 0.2866 nm	Ni: 0.3524 nm
Atomic Radius	Fe 0.126 nm	Ni 0.124 nm

**Table 2:** The model parameters of Fe-Pb and Ni-Pb.

	x	y	z	Sizes (Å)	Atom Number
Fe(001)-Pb	(100)	(001)	(010)	80 × 115.63 × 80	38,875
Fe(110)-Pb	(1-10)	(110)	(001)	80 × 115.76 × 80	39,435
Fe(111)-Pb	(-110)	(111)	(11-2)	80 × 117.06 × 80	38,635
Ni(001)-Pb	(100)	(001)	(010)	82.32 × 118.90 × 82.29	43,175
Ni(110)-Pb	(1-10)	(110)	(001)	81.27 × 117.75 × 82.19	40,875
Ni(111)-Pb	(-110)	(111)	(11-2)	81.27 × 122.77 × 81.95	43,691

**Table 3:** The interplanar distances of (001), (110), and (111) of bcc Fe and fcc Ni.

	d_(001)_	d_(110)_	d_(111)_
bcc Fe	1.4 Å	2 Å	0.8 Å
fcc Ni	1.8 Å	1.2 Å	2 Å

## Data Availability

The original contributions presented in this study are included in the article. Further inquiries can be directed to the corresponding authors.
